# Magnetic bead embedded in abdominal cavity successfully retrieved by endoscopy

**DOI:** 10.1055/a-2533-2922

**Published:** 2025-03-03

**Authors:** Zhihao Chen, Zhiling Li, Yue Li, Wanwei Liu, Dahui Huang, Min Yang, Zhigang Zeng

**Affiliations:** 1 89346 Department of Gastrointestinal Surgery, Guangdong Provincial Peopleʼs Hospital (Guangdong Academy of Medical Sciences), Southern Medical University, Guangzhou, China; 2 89346 Department of Pediatrics, Guangdong Provincial Peopleʼs Hospital (Guangdong Academy of Medical Sciences), Southern Medical University, Guangzhou, China; 3 89346 Department of Endoscopy, Guangdong Provincial Peopleʼs Hospital (Guangdong Academy of Medical Sciences), Southern Medical University, Guangzhou, China

A 2-year-old boy presented with loss of appetite and recurrent cough with expectoration for 1 year. Abdominal radiography performed 3 months prior revealed a circular, bead-like high-density shadow in the upper abdomen. Eleven magnetic beads were successfully extracted via gastroscopy at a local hospital; however, subsequent abdominal X-ray indicated that one bead remained in a fixed position ( Fig. 1 ).

**Fig. 1:**
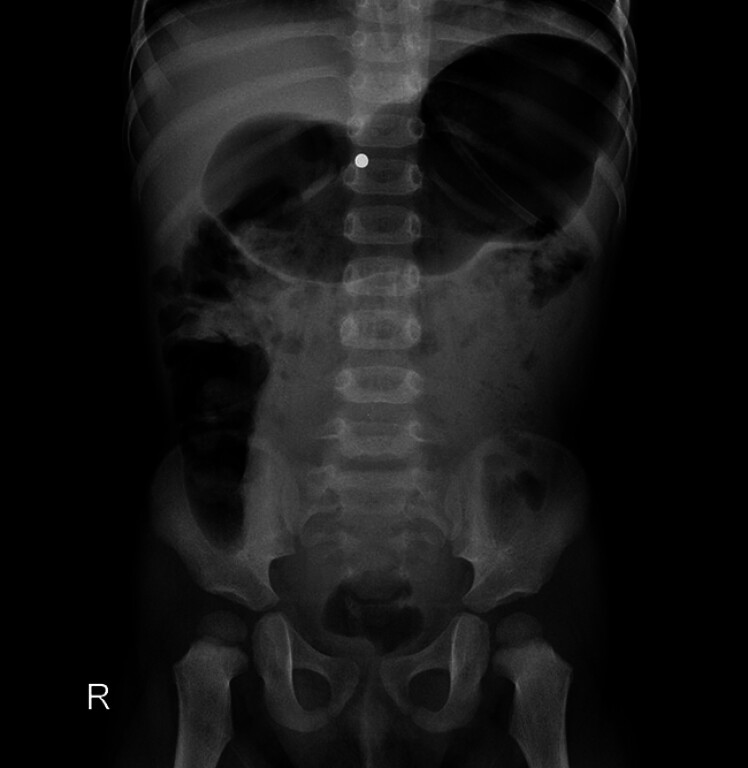
Abdominal X-ray showing a magnetic bead inside the body.

Upon admission to our hospital, gastroscopy revealed a scar on the lesser curvature of the antrum, but no foreign body was visible within the stomach ( Fig. 2 ). A subsequent endoscopic ultrasound identified a hyperechoic image outside the gastric wall at the site of the antral scar ( Fig. 3 ).

**Fig. 2:**
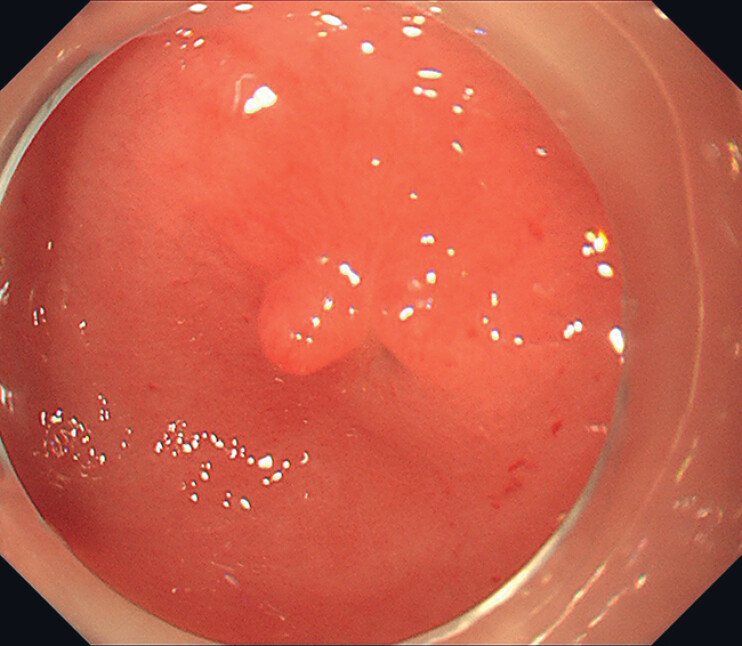
Gastroscopy showing a scar on the lesser curvature of the antral region.

**Fig. 3:**
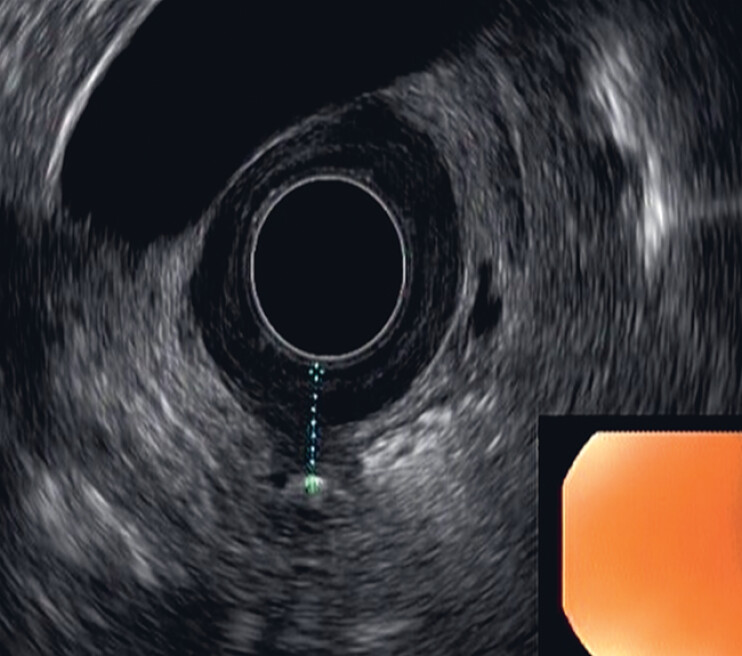
Ultrasound showing a strong echoic image outside the gastric wall at the site of the antral scar.

Following multidisciplinary team consultation, endoscopic full-thickness resection (EFTR) was attempted to remove the magnetic bead while minimizing surgical trauma. However, the bead could not be visualized during the procedure. To facilitate localization, a custom-made magnetic guidance device was employed. A small magnet embedded within an endoscopic retrieval net was introduced into the gastric cavity to detect the bead’s position ( Fig. 4 ). Once fully exposed, the bead was retrieved using endoscopic grasping forceps, and the wound was closed using hemostatic clips ( Video 1 ).

**Fig. 4:**
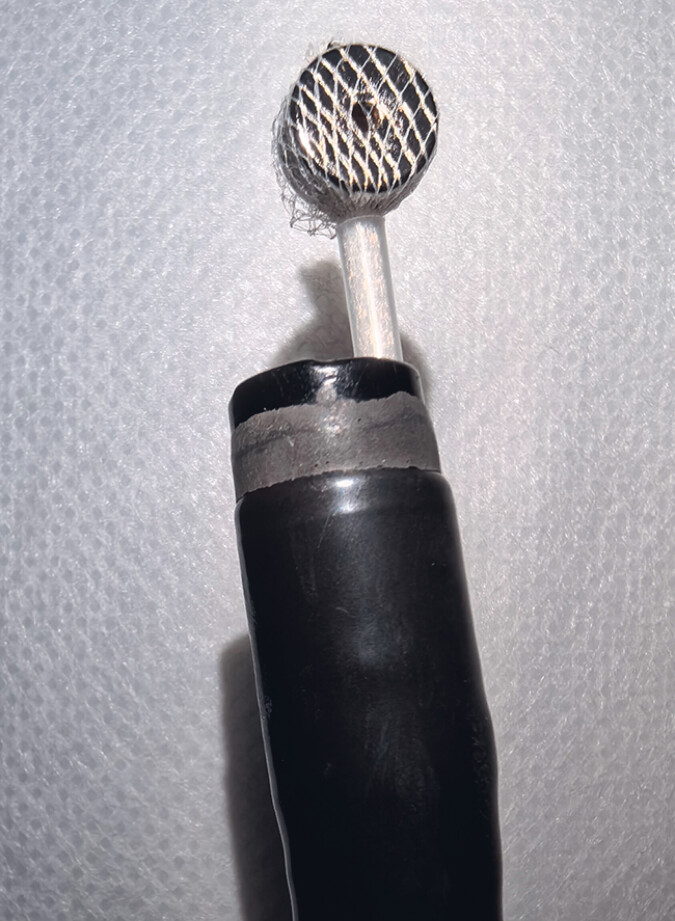
Custom-made guidance device featuring a small magnet embedded in a disposable endoscopic retrieval net.

A small magnet was embedded in an endoscopic retrieval net and introduced into the abdominal cavity to localize and remove a magnetic bead from a 2-year-old child.Video 1

The magnetic beads likely formed a cluster within the stomach due to their mutual attraction, potentially leading to transmural migration and encapsulation outside the gastric wall. EFTR, combined with the magnetic guidance device, successfully enabled foreign body removal without the need for invasive surgery.

The management of magnetic foreign body ingestion in children has been extensively reported 1 2 3 . However, to our knowledge, this is the first documented case of a magnetic bead embedded within the abdominal cavity of a 2-year-old child, successfully retrieved using an endoscopic approach alone. Accurate diagnosis is crucial, and our innovative endoscopic technique provides a minimally invasive alternative for similar cases.

Endoscopy_UCTN_Code_TTT_1AO_2AB

## Notes

Endoscopy E-Videos https://eref.thieme.de/e-videos
*E-Videos* is an open access online section of the journal *Endoscopy* , reporting on interesting cases and new techniques in gastroenterological endoscopy. All papers include a high-quality video and are published with a Creative Commons CC-BY license. Endoscopy E-Videos qualify for HINARI discounts and waivers and eligibility is automatically checked during the submission process. We grant 100% waivers to articles whose corresponding authors are based in Group A countries and 50% waivers to those who are based in Group B countries as classified by Research4Life (see: https://www.research4life.org/access/eligibility/ ). This section has its own submission website at https://mc.manuscriptcentral.com/e-videos .
